# Psychedelics action and schizophrenia

**DOI:** 10.1007/s43440-023-00546-5

**Published:** 2023-10-30

**Authors:** Marzena Maćkowiak

**Affiliations:** Laboratory of Pharmacology and Brain Biostructure, Pharmacology Department, Maj Institute of Pharmacology, Polish Academy of Sciences, Kraków, Poland

**Keywords:** Hallucinogens, 5-HT_2A_ receptor, Mental disorder, Therapy

## Abstract

Psychedelics are compounds acting by serotonin 5-hydroxytryptamine (5-HT)_2A_ receptor activation and induce several behavioral responses. They are of special interest because of their positive effects on neuropsychiatric disorders (depression and posttraumatic stress disorder). However, several findings revealed that some psychedelic actions are similar to symptoms observed in schizophrenia (psychosis, sensorimotor gating impairments, attention, and working memory deficits) which might limit their clinical applications. Psychedelics activate some neurotransmitters, i.e., serotonergic, and glutamatergic, that are also impaired in schizophrenia. Therefore, the neurobiological background of psychedelics and schizophrenia is partially similar. Another important aspect to discuss is the perspective of using psychedelics in schizophrenia therapy. Postmortem studies showed a loss of synapses in schizophrenia, and the positive effects of psychedelics on neuroplasticity (synaptogenesis, neurogenesis, and neuritogenesis) might be essential in the context of schizophrenia therapy. However, because of psychedelics' psychotic action, the recommended doses of psychedelics in schizophrenia treatment are not established, and subpsychedelic dosing or microdosing are considered. Exploratory studies are needed to determine the tolerability of treatment and appropriate dosing regimen. Another therapeutic option is using non-hallucinogenic psychedelic analogs that also induce neuroplastic outcomes but do not have psychotogenic effects. Further preclinical and clinical studies are needed to recognize the potential effectiveness of 5-HT_2A_ agonists in schizophrenia therapy.

## Introduction

Psychedelics (mind-manifesting) are psychoactive substances that induce alterations in mood, thought processes, perceptions, and experiences rarely experienced except in dreams, contemplative and exaltation, and acute psychosis (known as psychedelic experiences or psychedelic trips). They are also called psychotomimetic (psychosis-mimicking) or psycholytic (psyche-loosing) [1]. Most psychedelics derive from plants or semisynthetic and can be classified by chemical structure into indolamines and phenethylamines. The indolamine group includes lysergic acid diethylamide (LSD), semisynthetic ergosterol that naturally occurs as ergot alkaloid lysergic acid in the rye parasite; psilocybin, a compound found in magic mushrooms, *N,N-*dimethyltryptamine (DMT), the active ingredient in ayahuasca; 5-methoxy-*N,* *N-*dimethyltryptamine (5-MeO-DMT). The phenethylamine group includes mescaline, the active ingredient in the peyote and Sant Pedro cacti, and 2,5-dimethoxy-4-iodoamphetamine (DOI) [2].

The subjective effects of psychedelics have been assessed using the Hallucinogen Rating Scale (HRS) and Abnormal Mental States (APZ) questionnaire to measure altered states of consciousness (ASC) [3]. HRS elements were placed into six conceptually coherent clusters: somaesthesia, affect, perception, cognition, volition, and intensity. ASCs are defined by three primary dimensions: oceanic boundlessness (OSE, OBN, and OB), dread of ego dissolution (AIA, DED, and AED), and visionary restructuralization (VUS, VRS, and VR). The first dimension (oceanic boundlessness) measures derealization and depersonalization phenomena associated with positive basic mood. The second dimension (dread of ego dissolution) measures thought disorder, ego disintegration, and loss of autonomy and self-control associated with arousal, anxiety, and paranoid feelings. The third dimension (visionary restructuralization) is related to auditory and visual illusions, hallucinations, synaesthesias, and alterations in the meaning of various percepts [4]. ASC scale was revised to OAV scales, and later to the five-dimensional altered states consciousness (5AD-ASC) [3].

Psychedelics are classic serotonergic hallucinogens acting as an agonist or a partial agonist of the serotonin 5-hydroxytryptamine (5-HT)_2A_ receptor. Pharmacological studies showed that psychedelic effects in humans depend on the activation of 5HT_2A_ receptors, but not dopamine D_2_ receptors. Pretreatment with ketanserin, an antagonist of 5-HT_2A/2C_ receptors, effectively blocked the effects of psilocybin on all three dimensions of ASC. Likewise, pretreatment with an atypical antipsychotic, a mixed 5-HT_2A/D2_ antagonist, risperidone attenuated APZ scores in the psilocybin study, but no effect was observed after administration of a typical antipsychotic, haloperidol, a D_2_ receptor antagonist [5]. Neuroimaging studies in humans also showed significant psilocybin 5-HT_2A_ receptor occupancy in the brain that correlated with the psychedelic experience [6].

### 5-HT_2A_ receptors

5-HT_2A_ receptor is mainly expressed in the frontal cortex of humans and rodents, and it is also detectable at relatively lower densities in other brain regions, i.e., hippocampus, thalamus, and basal ganglia. In the cortex, 5-HT_2A_ receptors are predominantly found on the dendrites of excitatory glutamatergic pyramidal neurons [7–9].

There are known several 5-HT_2A_ receptor-coupled signaling pathways. The best-characterized signaling pathway is G_q/11_-mediated activation of a cytoplasmic protein phospholipase C (PLC) that cleaves phosphatidylinositol 4,5-biphosphate (PIP_2_), a phospholipid in the plasma membrane, followed by a generation of diacylglycerol (DAG) and inositol triphosphate (IP_3_). IP_3_ evokes the release of CA^2+^ from the endoplasmic reticulum, and the stimulation of protein kinase C (PKC) [10–13]. The 5-HT_2A_ receptor can also bind to other G proteins and downstream effector pathways. The 5-HT_2A_ receptor agonists stimulate phospholipase A_2_ (PLA_2_), and the release of arachidonic acid (AA). The activation of PLA_2_ is not dependent on *G*_q/11_ stimulation but correlates with *G*_i/o_ pathway and Src, and *G*_12/13_ activation of Rho [14–16]. Activation of the 5-HT_2A_ receptor also induces *G*_i/o_-dependent *Gβγ*-associates stimulation of extracellular signal-regulated kinase, p44/p42 (ERK1/2) [17]. There are several additional pathways linked to 5-HT_2A_ receptor stimulation, i.e., pERK through *β*-arrestin [18] or phospholipase D (PLD) by the small *G*-protein ADP-ribosylation factor (SRF) activation [19].

Several findings indicate that hallucinogenic responses caused by 5-HT_2A_ receptor agonists depend on the activation of specific signaling pathways. In vitro study demonstrated that hallucinogenic and non-hallucinogenic 5-HT_2A_ receptor agonists stimulate PLC signaling pathways, while hallucinogen-dependent reactions also include pertussis toxin (PTX)-sensitive heterotrimeric *G*_i/o_ proteins [20]. The results from in vivo and in vitro studies indicate that hallucinogens selectively activate *G*_i/o_-dependent signaling, whereas non-hallucinogenic 5-HT_2A_ agonists do not stimulate *G*_i/o_ [21].

Biased agonism of the 5-HT_2A_ receptor was also observed at the transcription level. Both hallucinogenic and non-hallucinogenic 5-HT_2A_ agonists induced c-Fos and IκBα transcripts. However, transcripts Egr-1 and Egr-2 were activated only by hallucinogens (DOI, DOM, DOB, mescaline, LSD, psilocin), but not by non-hallucinogenic 5-HT_2A_ agonists (R-lisuride, S-lisuride, ergotamine) [20, 22]. Hallucinogen-characteristic transcriptome fingerprints were dependent on the modulation of both *G*_q/11_ and *G*_i/o._ Recent findings indicate that hallucinogenic-like behavioral responses to 5-HT_2A_ agonists (head twitch in animals) seem to be related to the activation of *G*_q_ and *G*_s_ pathways. Hallucinogen agonists (DOM, 25C-NBOH) activate both *G*_q_ and *G*_s_ proteins and produce a head twitch response. In contrast, non-hallucinogenic agonists (lisuride and TBG) only activate G_q_ signaling and do not induce hallucinogenic-like behavior [23].

The above observation suggests that in the case of the 5-HT_2A_ receptor, a biased agonism related to the diverse signal transduction pathways stimulated by 5-HT_2A_ receptor agonists is detected. Thus, the distinct behavioral and molecular responses produced by hallucinogenic and non-hallucinogenic 5-HT_2A_ receptor agonists acting by the same population of cortical pyramidal 5-HT_2A_ receptors might be explained by that phenomenon.

### Schizophrenia

Schizophrenia is a severe, chronic disorder with symptoms divided into three groups: positive symptoms (delusions, hallucinations, inexplicable behavioral changes, and thought disorders), negative symptoms (avolition, anhedonia, asociality, blunted affect, and alogia), and cognitive deficits (poor learning, attention and working memory impairments, and retention of verbal information) [24–26].

Several findings indicate that schizophrenia is a neurodevelopmental disorder with first symptoms observed in late adolescence or young adulthood. The age of schizophrenia appearance depends on sex. In the case of men, the first signs are observed around the late teenagers to early 20s, in women between 20s and early 30s [27]. The epidemiological studies suggest the beginning of negative symptoms approximately five years before the initial psychotic episode, and the positive symptoms start close to the first hospitalization [28, 29].

The etiology of schizophrenia is still under investigation. However, epidemiologic and genetic evidence suggests that disease development results from the interaction of genetic background with environmental conditions. The above factors affect brain maturation during early life and adolescence [30]. Moreover, impaired epigenetic mechanisms, interacting with environmental risk factors and controlling gene expression, are also considered in the etiology of schizophrenia [31].

The pathophysiology of schizophrenia is still being explored. However, several neuroanatomical and neurotransmitter abnormalities are detected in schizophrenia. Neuroanatomical studies showed no changes in the total number of neurons in schizophrenia but an increase in neuronal density and a reduction in the neuropil were detected. Shorter dendritic length, lower dendritic spines on pyramidal neurons, and a decrease in the level of synaptophysin, a marker of axon terminals, and lower synaptic vesicle density are also noticed [32–36]. Some evidence shows 60% synapse loss in schizophrenia [37]. The schizophrenia hypothesis suggests that overpruning in synapses, mainly glutamatergic inputs onto cortical interneurons, disrupts excitation/inhibition (*E*/*I*) balance resulting in negative and cognitive symptoms development [38].

The above hypothesis is supported by neuroanatomical changes implying dysfunction in neurotransmitters, mainly excitatory ones. Alterations in the glutamate signaling might be related to the dysfunction of glutamatergic receptors i.e., the *N*-methyl-d-aspartate (NMDA) receptor, *α*-amino-3-hydroxy-5-methyl-4-isoxazolepropionic (AMPA) receptor, or metabotropic (mGlu) receptor. Genetic studies specified genes coding glutamate receptor subunits, GRIN2A for NMDA receptor, GRIA1, GRIA3 for AMPA receptor, or GRM3 for mGlu3 receptor as schizophrenia risk genes [39]. Moreover, the hypofunction of the NMDA receptor is postulated based on the effects of non-competitive NMDA receptor antagonists, ketamine, and phencyclidine (PCP). They were originally developed as dissociative anesthetics, but they are known to induce schizophrenia-like abnormalities in healthy humans. The symptoms include not only positive ones (psychosis, thought disorder) but also negative and cognitive deficits [40]. Antipsychotic effects of drugs acting selectively through glutamatergic signaling have been explored. Classical agonists of the NMDA receptor cause excitotoxicity and neuron damage, and they are not considered in schizophrenia treatment However, stimulation of NMDA receptor indirectly by glycinergic (serine, cycloserine) acting as NMDA coagonists and glycine transport (GlyT1) inhibitors (bitopertin) have been studied. In clinical trials, they demonstrate some effects on negative symptoms of schizophrenia [41]. Positive allosteric modulators of AMPA or mGlu receptors and mGlu agonists are also promising targets for new antipsychotic drugs. However, they are still under investigation and need evaluation in clinical trials [42, 43].

Another hypothesis proposes that disinhibition of excitatory cortical projection induces dysregulation of mesostriatal dopamine neurons and psychotic symptoms [38]. Positive symptoms result from hyperactivity in dopaminergic transmission in limbic pathways, while negative symptoms are thought to arise from hypodopaminergic functioning in the frontal structures. These interpretations are supported by the fact that all antipsychotics have an affinity to the D_2_ receptor and most of them are antagonists to this receptor [44]. Moreover, gene-associated studies showed a possible relationship between the risk of schizophrenia and some variants in genes related to dopamine signaling, i.e., catechol-O-methyltransferase (COMT), monoamine oxidase (MAO), dopamine transporter (SLC6A3), and dystrobrevin-binding protein 1 (DTNBP1) [39].

The role of serotonin, the especially 5-HT_2A_ receptor in schizophrenia pathogenesis is also considered. This hypothesis is based on the fact that psychedelics causing psychosis are agonists of the 5-HT_2A_ receptor. On the other hand, 5-HT_2A_ antagonists might have antipsychotic properties, mainly in negative symptoms [44]. Alternative 5-HT targets are also investigated in the context of schizophrenia treatment, i.e., 5-HT_1A_ agonists, 5-HT reuptake inhibitors, 5-HT_2C_ antagonists and agonists, 5-HT_3_ antagonists, 5-HT_6_ antagonists, 5-HT_7_ antagonists. However, there are still no drugs with selective serotonin pharmacological profile used in schizophrenia therapy [41].

Postmortem studies examining the level of 5-HT_2A_ protein showed inconsistent results. The [^3^H] ketanserin binding study revealed a decrease in the density of 5-HT_2A_ receptors in cortical areas of schizophrenia patients [45]. However, another [^3^H] ketanserin binding examination reported an increase in the number of [^3^H] ketanserin binding sites to the 5-HT_2A_ receptor in the postmortem prefrontal cortex of antipsychotic-free but not in antipsychotic-treated schizophrenic subjects [46]. On the other hand, a western blot analysis showed no changes in 5-HT_2A_ receptor immunoreactivity in the prefrontal cortex in either treated with antipsychotics or not treated schizophrenia patients but a decrease in mRNA level was found only in patients treated with antipsychotics [47]. The above findings indicate that the level of the 5-HT_2A_ receptor in schizophrenia might depend on antipsychotic therapy but also on methods used in studies. Thus, the role of the 5-HT_2A_ receptor in schizophrenia pathomechanism is not definitively defined yet.

### Schizophrenia-like effects of psychedelics

Psychedelics induce schizophrenia-like related symptoms i.e., psychosis, sensorimotor gating impairments, and working memory deficits (Table 1).

**Table 1:** Comparison between schizophrenia and psychedelic effects

Abnormalities	Psychedelics	Schizophrenia	References
Behavioral response			
Psychosis	Mainly visual hallucinations, geometric, complex, reality assessment present, transient psychotic episodes, lasting a few hours	Visual hallucinations in the acute stage life-size images, real, detailed, anchored in space, auditory hallucinations in the chronic phase, poor reality monitoring, recurrent psychotic episodes lasting weeks or months	[4, 50]
	Existential/metaphysical meaning	Existential/metaphysical meaning	
Sensorimotor gating	Mainly impaired, depending on psychedelics	Impaired	[61–63, 70–73]
Working memory	Impaired	Impaired	[5, 75, 76, 79–83]
Neurochemical background			
Neuronal activity	Hyperfrontality	Hyperfrontality in acute episodes, hypofrontality in the chronic phase	[49, 51–59]
5-HT_2A_ receptor	Activation: hallucinogens G_q11_ and G_i/o_ pathways, non-hallucinogens G_q11_ pathway	polymorphism, inconsistent data related to 5-HT_2A_ level	[20–22, 45–47, 67, 68, 77, 78]
Glutamatergic transmission	5-HT_2A_–mGlu2 complex, indirect activation of NMDA, AMPA receptors	Genetic predisposition, NMDA hypofunction	[39, 40, 84, 85, 95]

### Psychosis

Psychosis is a clinical syndrome represented by several symptoms i.e., delusions, hallucinations, and thought disorder [48]. Psychosis is a common feature of both psychedelic actions in normal volunteers and schizophrenia patients.

Psychedelics induce a psychosis-like syndrome in normal volunteers that is characterized by ego disturbances, illusions and hallucinations, thought disorders, paranoid thinking, and alterations in mood and affect [4, 5, 49]. Several psychotic symptoms induced by psychedelics are similar to the positive symptoms observed in the initial stages of acute schizophrenia episodes. Hallucinations and the loss of self-control over thought processes after the administration of psychedelics are similar to those experienced in acute psychosis in schizophrenia. Moreover, visual hallucinations typical for psychedelics are more often in acute than chronic schizophrenia [4]. However, hallucinations observed either in healthy volunteers using psychedelics or in patients with schizophrenia differ in many respects. Hallucinations induced by psychedelics are mostly visual, and both elementary (brightly colored geometric form figures) and complex (images of scenes and landscapes) with ordinary (humans, animals, and artifacts) and extraordinary entities (chimeras, spirits, and aliens). In schizophrenia, hallucinations mostly auditory, and visual hallucinations often include life-size images (faces, people, objects, events), and they are real, detailed, and anchored in space. In both cases, hallucinations have strong existential/metaphysical meanings. Usually, reality assessment is not impaired in people using hallucinogens, and they can distinguish between psychedelic effects and normal consciousness. In contrast, patients with schizophrenia have poor reality monitoring and insight. The duration of psychotic episodes is also different. Psychedelics induce transient psychotic episodes lasting a few hours unlike in schizophrenia patients, where the psychotic episodes are recurrent, and can last weeks or months [50].

Neuronal activity during psychosis was examined using positron emission tomography (PET) and [F-18]-fluorodeoxyglucose (FDG) that can analyze the organization of the human brain with neuronal function [51]. The results of the studies showed metabolic hyperfrontality during psychotic episodes in healthy volunteers using psychedelics (psilocybin, mescaline) [49, 52]. Hyperfrontal metabolic pattern was also associated with positive psychotic symptoms in acute unmedicated first-episode schizophrenia [53, 54] and also in unmedicated and medicated chronic patients with acute psychotic episodes [55, 56]. Thus, hyperfrontality metabolic changes are associated with acute psychotic episodes in schizophrenics and they are also induced by psychedelics, with contrasts to hypofrontality observed in patients with chronic schizophrenia [57–59].

### Sensorimotor gating

Sensorimotor gating ability regulates sensory information that is transmitted to motor output systems. Sensory information processed centrally requires some degree of filtering or gating before accessing and impacting motor output [60]. Prepulse inhibition (PPI) of the acoustic startle response has been established as an operational measure of sensorimotor gating, and the PPI level may indicate the current integrity of sensorimotor gating mechanisms. PPI occurs when a relatively weak sensory stimulus (prepulse) is presented 30–500 ms before a strong startle-induced stimulus (pulse), and reduces the magnitude of the startle response. The fundamental mechanism initiating this inhibition is thought to resemble the normal process of filtering incoming sensory stimuli [61]. In humans, startle is measured from the eye blink response through electromyographic recordings from the orbicularis oculi muscle, and startle to acoustic stimuli or tactile stimuli are used [60]. PPI deficits are observed in schizophrenia patients [61–63], their unaffected relatives [64], and patients with schizotypal personality disorder [65].

Sensorimotor gating functions are modulated by several neurotransmitter systems i.e., dopaminergic, glutaminergic, serotonergic, γ-aminobutyric acid (GABA)-ergic, and cholinergic acting in cortical, limbic, striatal and brainstem structures [61, 66]. Some studies indicate that, among others, 5-HT_2A_ serotonin receptors are involved in proper sensorimotor gating and 5-HT_2A_ receptor polymorphisms might contribute to the PPI deficits in schizophrenia [67, 68]. Analysis of 5HT_2A_ receptor polymorphism (A-1438G, T102C, and H452Y) showed that patients carrying the T102CTT and the A-1438GAA allele demonstrated higher PPI levels than all other variants. Carriers of the T102C-C/A-1438G-G allele exhibited a significantly lower PPI than patients homozygous for the T102C-T/A-1438G-A allele. On the other hand, the 5-HT_2A_ receptor H452Y polymorphism did not affect startle parameters [68]. Another evidence indicates that epigenetic modifications (DNA methylation) of the 5-HT_2A_ receptor allele are also involved in 5-HT_2A_ receptor regulation, especially in an early stage of disease onset [67]. Thus, genetic background and epigenetic mechanism might implicate 5-HT_2A_ receptor function in sensorimotor gating.

Hallucinogens are postulated to work by disrupting sensory filtering mechanisms, resulting in sensory overload and cognitive dysfunction [4, 69]. The effects of psychedelics on PPI were analyzed in humans and obtained results revealed diverse effects of psychedelic substances on PPI. DMT did not affect PPI at any using interstimulus intervals (ISI) [70]. In the case of psilocybin, some studies show an increase in PPI at 100 ms ISI [71], while others indicate that the effect of psilocybin on PPI was dependent on ISI. Psilocybin reduced PPI at short (30 ms), had no effect at medium (60 ms), and increased PPI at long (120–2000 ms) intervals [72]. On the other hand, LSD did not demonstrate a similar to psilocybin impact on PPI and decreased PPI at 30 ms, 60 ms, and 120 ms ISI [73]. Thus, hallucinogens alter PPI in humans, but the effects depend on the used substance, and ISI applied in the studies.

### Working memory

Working memory is a limited-capacity, active short-term memory system that maintains information to guide and control behavior [74]. Working memory deficits are a permanent cognitive feature of schizophrenia and memory impairments are present during the prodromal stage and persist throughout schizophrenia [74]. Spatial working memory deficits were observed in schizophrenic patients in a spatial delayed-response task (DRT) [75]. Working memory was also studied in the Sternberg Item Recognition Test (SIRT), and functional magnetic resonance imaging (fMRI) showed that memory impairment in schizophrenic patients was related to a reduction in information processing efficiency, especially in the dorsal prefrontal cortex, or lack of an increase fMRI activation during task presentation was leading to performance impairments [76].

Functional genetic variants have a specific impact on cognitive abilities in a normal population. A study of two polymorphisms (rs6313 and rs4941573) in the 5-HT_2A_ receptor showed that rs4941573 was associated with spatial working memory [77]. Some studies indicate that polymorphism in the T102C and A-1438G loci of the 5HT_2A_ receptor is correlated with working memory impairment in schizophrenia patients, and T102C CC and A-1438G GG homozygotes have worse performance in working memory tasks [78].

Working memory was also examined in healthy participants using hallucinogens acting by 5-HT_2A_ receptor (psilocybin and LSD). Psilocybin (0.25 mg/kg) induced spatial memory deficits in humans as measured by DRT. These deficits were linked to the activation of 5-HT_2A_, but not D_2_ receptors [5]. Another finding reported that psilocybin (215 μg/kg) reduced attentional tracking ability analyzed in a multi-object tracking test but did not affect spatial working memory measured in the Spatial Span test from Cambridge Neuropsychological Test Automated Battery (CANTAB) [79]. Ketanserin did not block the effect of psilocybin on attentional performance suggesting an involvement of the serotonin 5-HT_1A_ receptor in the observed deficits [79]. The effect of psilocybin in medium 115 μg and high 250 μg/kg dose conditions on spatial working memory in the Spatial Span test was also examined by others [80]. Significant impairments in performance on the task were observed in the high-dose condition but not in the medium-dose condition. Working memory was also assessed with the letter *N*-back task from Penn Computerized Neurocognitive Battery, and psilocybin (10, 20, and 30 mg/70 kg) dose-dependent impaired the performance in the N-back test [81]. The above results indicate that the effect of psilocybin on working memory might be dependent on the dose used in the studies. Another psychedelic, LSD (100 μg) impaired working memory examined by Intra/Extra-Dimensional shift task (IED) and Spatial Working Memory task (SWM) from CANTAB [82]. Ayahuasca users also showed impairments in working memory performance tested in the Stenberg working memory task, and the Tower of London task [83].

Recent findings indicate that polymorphism of the 5-HT_2A_ receptor as well as activation of the 5-HT_2A_ receptor by psychedelics might induce the disruption of working memory that is observed in schizophrenia patients.

### Glutamatergic transmission in psychedelics action

Numerous animal studies showed that the effects of psychedelics might be related to other than serotonergic transmission (Table 1). Early findings indicate that immediate-early genes (IEGs), i.e., c-Fos, Arc proteins induced by psychedelics, i.e., DOI, are not expressed in the 5-HT_2A_-positive cells [9, 84, 85]. The observed results of DOI action were directly relatable to the activation of 5-HT_2A_, but not the 5-HT_2C_ receptors [84, 85]. Later studies showed that psychedelics (LSD and DOI) evoked c-Fos expression is present in cells positive for 5-HT_2A_ mRNA [20], but the lack of 5-HT_2A_ immunoreactivity in cells demonstrating DOI-stimulated IEGs transcription might suggest an indirect effect of hallucinogens on IEGs activation.

Dysregulation of glutamatergic signaling is indicated to be involved in schizophrenia pathogenesis [40], and some evidence also implies that glutaminergic transmission is involved in the hallucinogenic actions following 5-HT_2A_ receptor activation. A recent study showed that psilocybin increases glutamate release in the rat frontal cortex [86] and human medial prefrontal cortex [87]. Other findings indicated that either an antagonist of the AMPA receptor, GYKI 52466, or an antagonist of the NMDA glutamate receptor, MK-801, attenuated an increase in IEGs expression (Arc, c-Fos) induced by DOI [84, 85]. The above results imply that ionotropic receptor activation, i.e., NMDA, and AMPA, is necessary for transcriptional activities induced by psychedelics. Moreover, a recent study revealed that psilocybin increased the level of NMDA receptor subunits, mainly GluN2B, but it did not affect the protein of AMPA receptor subunits (GluA1, GluA2) in the rat frontal cortex [86]. The above findings indicate that ionotropic glutamate receptors might play an essential role in the action of psychedelics.

Electrophysiological studies revealed that enhancement of 5-HT_2A_ receptor-induced glutamatergic transmission in the prefrontal cortex was suppressed by activation of glutamatergic metabotropic receptor mGlu2/3 [88, 89] and an agonist of mGlu2/3 also reduced DOI-induced c-Fos expression [89]. The above findings suggest that the activation of presynaptic mGlu2 autoreceptors blocked the stimulation of glutamatergic transmission induced by hallucinogenic 5-HT_2A_ receptor agonists [90]. Another evidence implies that crosstalk between 5-HT_2A_ and mGlu2 receptors is related to their presence in the postsynaptic part of cortical pyramidal neurons. The G-protein coupled receptors (GPCRs) studies in mouse and human cortex showed that G_q11_-coupled 5-HT_2A_ and the G_i/o_ -coupled mGlu2 receptor form a specific GPCR heteromeric complex [20, 91, 92]. 5-HT_2A_-mGlu2 receptor complex seems to be essential for the hallucinogenic-like behaviors caused by 5HT_2A_ agonists i.e., head-twitch in animals. 5-HT_2A_ receptor agonist (DOI, LSD) did not induce head-twitch behavior in mGlu2 knockout mice [93]. Moreover, behavioral responses of 5-HT_2A_ receptor activation were returned in mice with overexpression mGlu2 in the frontal cortex using a viral (HSV)-mediated transgene expression method [92].

The results from animal studies have great translational potential since postmortem analysis of the frontal cortex of schizophrenic subjects revealed the changes in the expression of 5-HT_2A_ and mGlu2 receptors [46, 92, 94]. Moreover, functional crosstalk between 5-HT_2A_ and mGlu2 receptors was also found in schizophrenia because the activation of G_q11_ signaling by mGlu2 receptor agonist was dysregulated in the postmortem frontal cortex of schizophrenic subjects [94]. Thus, an interaction between 5-HT_2A_ and mGlu2 receptors could be a relevant explanation of the mechanism involved in psychosis and schizophrenia treatment [95].

### 5-HT_2A_ receptor as a target in schizophrenia treatment

#### 5-HT_2A_ receptor antagonists

Antipsychotics are a group of drugs used in schizophrenia therapy. They are divided based on data of release (first vs. second generation), efficacy, and side effects. However, all antipsychotics share a blockade of the D_2_ receptor as a necessary mechanism for their action [96]. They are predominantly useful in positive symptoms of schizophrenia and have serious limitations in therapy including low effectiveness for negative symptoms and cognitive deficits, and side effects (for example weight gain, metabolic disturbances, extrapyramidal symptoms (EPS), akathisia, hyperprolactinemia, and sleep disturbances) [97].

First-generation antipsychotics (chlorpromazine and haloperidol) are characterized by their relatively high-affinity antagonism at the D_2_ receptor, and they are efficacious on positive symptoms of schizophrenia. In contrast, most second-generation antipsychotics (clozapine, risperidone, and olanzapine) have relatively lower D_2_ receptor antagonism but they possess relatively higher 5-HT_2A_ receptor antagonism that is supposed to increase their efficacy in the treatment of negative and cognitive abnormalities in schizophrenia [97]. Recently, a new antipsychotic, lumateperone has been approved by the Drug and Food Administration (FDA). Lumateperone is an antagonist of 5-HT_2A_ and postsynaptic D_2_ receptors but also acts as a partial agonist of the presynaptic dopamine D_2_ receptor. Similar to other second-generation antipsychotics, it has a higher affinity for the 5-HT_2A_ receptor compared to the D_2_ receptor. Clinical studies reveal that lumateperone significantly reduces positive and negative schizophrenia symptoms and does not present EPS and metabolic adverse effects usually observed in antipsychotic treatment [98, 99]. Thus, lumateperone seems to be a promising option for schizophrenia therapy in patients with metabolic dysfunction and intolerance to EPS.

A meta-analysis study indicated that 5-HT_2A_ antagonists could be useful in the treatment of negative symptoms of schizophrenia [100]. Recent findings also suggest that the antipsychotic affinity for the 5-HT_2A_ receptor is not significantly associated with specific side effects i.e., sedation, EPS, prolactin increase, and weight gain [101].

Clinical use of selective antagonists of the 5-HT_2A_ receptor in schizophrenia pharmacotherapy has not been approved by FDA. 5-HT_2A_ antagonists, such as roluperidone (MIN-101), eplivanserin (SR46349B), fananserin, and ritanserin, were checked in clinical trials, however, their effectiveness in schizophrenia treatment was not satisfactory [97]. At this moment, there are no FDA-approved non-dopaminergic antipsychotics for schizophrenia therapy. On the other hand, pimavanserin, a reverse agonist of the 5-HT_2A_ receptor with affinity to 5-HT_2C_ but not the D_2_ receptor, is used in the treatment of hallucinations and delusions associated with Parkinson’s disease psychosis [97]. Moreover, combined treatment of pimavanserin with a subeffective dose of risperidone increased the therapeutic effects and enhanced the safety of risperidone treatment in schizophrenia [102]. Thus, the 5-HT_2A_ receptor might be a promising target in schizophrenia pharmacotherapy but an affinity and antagonism only to the 5-HT_2A_ receptor are not sufficient to get full antipsychotic effects.

#### 5-HT_2A_ receptor agonists

Hallucinogenic properties of 5-HT_2A_ agonists exclude schizophrenia from the current list of potential therapeutic targets of psychedelics. Given the historical background of the administration of psychedelics to schizophrenia patients in the 1950s and 1960s showing their psychotomimetic and hallucinogenic properties [103, 104], their clinical applications require extreme caution. However, in some studies from the 1950s and 1960s, psychedelic administration to young people with autism and schizophrenia improved symptoms domain called today as negative symptoms. What is more, exacerbation of psychosis was not always observed [105, 106].

At present, people with a personal family history of psychosis are generally excluded from clinical trials of psychedelic compounds [107]. However, there are some presumptions that 5-HT_2A_ agonists might be worthy of consideration in schizophrenia therapy, especially when negative symptoms and cognitive deficits are predominant in the features of the disorder [108], and these deficits are associated with structural brain changes, i.e., significant cortical thinning [109–111], reduction gray matter volume [112], reduction in synaptic proteins and synaptic loss [33, 113]. In the context of the above neuroanatomical changes observed in schizophrenia, the 5-HT_2A_ receptor agonist's ability to produce rapid and long-lasting changes in neural plasticity might be essential from the perspective of schizophrenia therapy. Several findings indicate that psychedelics (DOI, psilocybin, LSD, DMT) increase dendritic arbor complexity, promote dendritic spine growth and stimulate synapse formation [114–118]. The above alterations are related to the neurotrophic effects of psychedelics. They increase neurotrophic factor levels, i.e., brain-derived neurotrophic factor (BDNF) and activate its receptor TrkB [115, 119–122]. Neurotrophin levels are impaired in schizophrenia and antipsychotic therapy has diverse effects on their level [123]. Thus, psychedelic treatment might have positive outcomes on synapse loss and cognitive deficits observed in schizophrenia by normalizing neurotrophin levels.

Evidence indicates that the window of neuroplastic effects for psychedelics is between a few hours to a few days, although some neuroplasticity alterations might be observed for at least a month [124]. Psychedelics also induce long-lasting transcriptomic and epigenomic changes, and most of them are related to genes associated with synaptic plasticity (axonogenesis, synapse organization, synapse assembly). However, some of them are correlated with genetic risk for schizophrenia development (Cntnap2, Nfasc, Drd2, Grin2b, Prkca, Slc1a2) [114, 125, 126].

The potential benefits of using 5-HT_2A_ receptor agonists in schizophrenia treatment cause a discussion about strategies for avoiding the psychotogenic effects of psychedelics while maintaining neuroplastic effects. At the doses used in the therapy of psychiatric disorders, all psychedelic drugs induce the “trip”. The patients required observation up to 24 h after treatment [127]. Presently, the doses used in clinical studies with psilocybin are 20–25 mg/70 kg for adults selected to stimulate evidently, subjectively experienced effects on perception, cognition, and mood [107]. On the other hand, some findings indicate that psychedelics might be used in microdoses to get therapeutical effects [128, 129]. Psychodelic microdose is defined as 10% of the dose inducing psychedelic effects in the average adult, i.e., for psilocybin 2.0–2.5 mg [130]. Psychedelic microdosing could be operated in schizophrenia treatment without inducing psychotic effects. However, further studies will be needed to determine the appropriate dose and dosing regimen given therapeutic benefits in improving negative and cognitive symptoms with the absence of psychotogenic effects, and also tolerability of treatment [107].

Pharmacological studies showed that some 5-HT_2A_ agonists do not possess hallucinogenic properties [131] and they have different transcriptome fingerprints [22]. Moreover, some evidence indicates that non-hallucinogenic psychedelic analogs might be a therapeutic option because of their ability to induce neuroplastic effects without a trip [132–134]. However, more studies are needed to verify their usefulness in the treatment of negative or cognitive schizophrenia dysfunction.

Thus, psychedelic microdosing or non-hallucinogenic agonist administration might be considered an alternative therapeutic option in schizophrenia treatment. However, at present 5-HT_2A_ agonists are not used for treatment of patients with schizophrenia.

## Summary and conclusions

The studies of using psychedelics in clinical therapy of psychiatric disorders started in the 1950s and 1960s but revealed that psychedelic behavioral effects shared some similarities with schizophrenia symptoms. Subsequent biochemical studies in animal models indicated that there is a certain likeness between psychedelics and schizophrenia pathophysiological background, including 5-HT_2A_ receptor polymorphism in schizophrenia, and glutamatergic transmission dysfunction in both cases. However, in the case of psychedelics, an interaction between 5-HT_2A_ and mGlu2 receptors seems to play an important role in the hallucinogen's appearance, in schizophrenia hypofunction of the NMDA receptor is suggested. At present, there is a lot of data showing an opportunity to use psychedelics in therapy for several psychiatric disorders (depression or posttraumatic stress disorders), but their use in schizophrenia treatment is still uncertain. The neuroplastic effects of hallucinogens and non-hallucinogens 5-HT_2A_ agonists would be of interest in the treatment of negative and cognitive deficits in schizophrenia, and the safest option would be to use non-hallucinogenic ones. Currently, there is not enough data to predict the further application of 5-HT_2A_ agonists in schizophrenia treatment, although it might be a beneficial target for new therapies. Additional preclinical and clinical investigations are necessary to confirm the effectiveness of psychedelics in schizophrenia treatment.

## Data Availability

Data sharing is not applicable for this article as no datasets were generated or analyzed during the current study.

## Abbreviations

5-HT_2A_: Serotonin 5-hydroxytryptamine receptor
AMPA: *α*-Amino-3-hydroxy-5-methyl-4-isoxazolepropionic receptor
ASC: Altered states of consciousness
APZ: Abnormal Mental States
DMT: *N, N-*Dimethyltryptamine
DOI: 2,5-Dimethoxy-4-iodoamphetamine
HRS: Hallucinogen rating scale
IEG: Immediate-early gene
LSD: Lysergic acid diethylamide
mGlu: Metabotropic receptor
NMDA: *N*-Methyl-d-aspartate receptor
PPI: Prepulse inhibition of the acoustic startle response
