# Correction: Psychedelics action and schizophrenia

**DOI:** 10.1007/s43440-024-00631-3

**Published:** 2024-08-02

**Authors:** Marzena Maćkowiak

**Affiliations:** Laboratory of Pharmacology and Brain Biostructure, Pharmacology Department, Maj Institute of Pharmacology, Polish Academy of Sciences, Kraków, Poland

**Correction: Pharmacological reports (2023) 75:1350–1361**

10.1007/s43440-023-00546-5

In the sentence beginning ‘The indolamine group includes lysergic acid diethylamide (LSD)…’ in Introduction section, the text ‘the active ingredient in morning glories’ should have been deleted.

The original article has been corrected.

